# Rhythm-Motor Dual Task Intervention for Fall Prevention in Healthy Older Adults

**DOI:** 10.3389/fpsyg.2019.03027

**Published:** 2020-01-17

**Authors:** Soo Ji Kim, Ga Eul Yoo

**Affiliations:** ^1^Music Therapy Education, Graduate School of Education, Ewha Womans University, Seoul, South Korea; ^2^Department of Music Therapy, Graduate School, Ewha Womans University, Seoul, South Korea

**Keywords:** dual task, fall prevention, healthy elderly, instrument playing, music intervention

## Abstract

This study aimed to investigate the effects of a rhythm-motor dual task intervention on cognitive and gait control for older adults in relation to fall prevention. Ten healthy older adults participated in a rhythm-motor dual task intervention and 10 participated in the control group. The intervention group received 16 30-min intervention sessions for 8 weeks. During the intervention sessions, participants engaged in walking or bimanual tapping as a primary motor task with concurrent rhythm tasks including playing instruments and rhythmic chanting or singing. At pretest and post-test, measures of cognition, balance/mobility, and gait were administered. A significant difference between groups was found for part B of the Trail Making Test (TMT-B) measure that involved executive control of attention. Also, changes in the gait ratio in the dual task condition of walking while playing an instrument were significantly different between groups. The findings in this study support the use of the rhythm-motor dual task intervention for increasing available cognitive resources and improving gait control, which are critical factors in fall prevention.

## Introduction

The risk of falling increases with age, and falling among the elderly commonly results in decreased physical and social functioning, reduced cognitive performance, and lower quality of life (Karlsson et al., 2011). As a result, there are increasing calls for interventions that can prevent falls among older adults. Recent research indicates that any intervention targeting gait control for fall prevention should be based on the interplay between motor and cognitive functions (Amboni et al., 2013). This research demonstrates that gait involves cognitive resources and attentional control rather than being an automated motor task (Hausdorff et al., 2005). As aging interferes with such cognitive processing, interventions for older adults should focus on enhancing their ability to adapt to increased attentional loads while walking (Rubenstein, 2006).

As such, dual task performance, which refers to the ability to control two tasks simultaneously, is considered a critical factor for mediating gait control (Yogev-Seligmann et al., 2008). Since walking in everyday environments involves attention to concurrent stimuli (e.g., traffic signs and obstacles in a walkway), high dual task performance is associated with a stable and safe gait (Neider et al., 2011). Decreased dual task performance become more prominent with advanced age and result in slower gait, shorter steps, shorter stride length, and increased gait variability (Al-Yahya et al., 2011). Particularly, walking speed has been documented as a important predictor for falls (Beauchet et al., 2008) and maintaining a constant walking speed in a challenging environment (e.g., increased cognitive demand while walking) is important for a safe gait (Hak et al., 2013). While decreased walking speed during dual task performance is associated with decline in efficient attention control in some studies (Beauchet et al., 2008), increased walking speed explains decrease in gait control in other studies (van Iersel et al., 2006). These findings imply that not only whether walking speed increases or decreases, but also the way of gait control can be important factors for fall risks of older population. Also, this emphasizes that older adults need their own strategies to adapt their gait (Schaefer et al., 2015) and dual task interventions should be developed to enhance such strategies.

Previous studies demonstrate that dual task performance can be improved with training (Agmon et al., 2014). In fact, specified dual task training led to expected outcomes, while single task interventions showed limited generalization of intervention effects (Oswald et al., 2006; Silsupadol et al., 2009; Agmon et al., 2014). Dual task interventions were found to improve not only gait, but also cognitive performance. Improved dual task performance contributed to more efficient control of attention and task-shifting (Silsupadol et al., 2009), even with older adults with cognitive impairment (Crossley et al., 2004). Such outcomes eventually led to decreased falls and increased competency in balance and gait.

In terms of training effects, recent studies reported that different types of tasks generated differential outcomes (Chu et al., 2013; Agmon et al., 2014). Under the dual task paradigm, gait functioning was disturbed to a greater extent when the interference was internal, such as when recalling verbal information or doing arithmetic tasks, compared to external interference, such as when reacting to external stimuli (Al-Yahya et al., 2011). As such, the addition of a mental tracking task (i.e., holding information internally while performing the presented task, such as counting backward by 3 s) while walking significantly predicted falls (Chu et al., 2013). These findings highlight the importance of selecting appropriate tasks in consideration of their applicability for the target population given their level of cognitive decline.

Along with specificity and intensity, successful engagement in an intervention is dependent upon the participants’ adherence to the protocol. Given the limited range in which the difficulty of cognitive tasks can be adjusted, music-based interventions offer a more flexible alternative and can be applied to older populations with varying levels of cognitive impairment and motivation. The use of musical stimuli in cognitive and motor tasks has been shown to increase efficiency in information processing in older populations with cognitive aging (Peper et al., 2012; Terrier and Dériaz, 2013). In addition, the use of rhythmic cueing led to effective coordination of increased attentional loads (Peper et al., 2012). With rhythmic cueing, instrument playing could be applied for tasks involving executive control and cognitive flexibility in older populations (Kim et al., 2017b). Despite their potential, attempts to systematically investigate the effects of music-based dual task intervention remain limited.

Furthermore, despite the importance of dual task interventions for older adults, there have been few attempts to develop such interventions for this population, with the research focus mainly being on individuals with clinical diagnoses. There are calls to expand the usage of this intervention to populations in community settings. Therefore, this study aimed to investigate a rhythm-motor dual task intervention for fall prevention with healthy older adults.

## Materials and Methods

### Participants

All procedures and ethical issues related to this study were approved by the Institutional Review Board of Ewha Womans University (IRB No. 136-5). Older adults aged 65 years and over were recruited from local community centers. A written informed consent was obtained from each participant. A total of 30 female older adults were recruited, and 16 of them were randomly assigned to the intervention group and 14 to the control. Six participants withdrew from the intervention group and four from the control group due to illness (*n* = 2), unwillingness to continue participation in the study (*n* = 4), and failure to participate in the post-test (*n* = 4). As a result, 20 female older adults (10 for the intervention group and 10 for the control group) were included in the final analysis. The Korean versions of the Mini-Mental State Examination (MMSE) and Geriatric Depression Scale (GDS) were used to screen for the inclusion criteria for participants. Individuals were included if they obtained a score of 27 or higher on the MMSE and if they scored less than 16 on the GDS. Participants’ demographic information is displayed in Table 1.

**TABLE 1:** Demographic information of participants.

**Parameter**	**Intervention (*n* = 10)**	**Control (*n* = 10)**
Sex, M:F	0:10	0:10
Age, years (*M* ± *SD*)	78.8 ± 7.8 (range: 67–90)	70.2 ± 3.9 (range: 65–76)
Education, years (*M* ± *SD*)	12.3 ± 4.3	6.6 ± 1.9
Falling after 60, *n*(%)		
Never	4 (40%)	8 (80%)
Once	3 (30%)	2 (20%)
More than twice	3 (30%)	0 (0%)
Falling in past 6 months, *n*(%)		
Never	8 (80%)	10 (100%)
Once	1 (10%)	0 (10%)
More than twice	1 (10%)	0 (0%)
MMSE (*M* ± *SD*)	28.8 ± 1.1	28.1 ± 1.5
GDS (*M* ± *SD*)	3.6 ± 4.5	6.0 ± 6.1

*MMSE, Mini-Mental State Examination; GDS, Geriatric Depression Scale.*

### Rhythm-Motor Dual Task Intervention

For this study, the rhythm-motor tasks involved walking and bimanual tapping as the primary motor task. While the use of walking paralleled the functional movements necessary in everyday life that put the participant at risk for falling, we also incorporated bimanual tapping for training attentional control at an intensive level while utilizing resources advantageous for older populations. Previous research points out that bimanual coordination in response to external timing cues, particularly involving two limbs in opposite directions (e.g., tapping in alternation), are associated with dynamic control of attention and motor output, which are critical for dual task performance (Kim et al., 2017b). Accordingly, this study included simultaneous/alternative bimanual tapping, or a combination of the two as the primary motor task as well as walking.

The concurrent rhythm task included playing instruments with different rhythm patterns using both hands and rhythmic chanting or singing (see Table 2). Instruments were used to require coordinated involvement of both hands, such as djembes, claves, and woodblocks. For familiar songs used, traditional songs, popular Korean songs, and popular English songs were included to allow for consideration of each participant’s musical preferences. During the intervention, the consistent use of rhythmic cueing via live accompaniment of regularly paced rhythm patterns or the concurrent use of a metronome aimed to facilitate adjustment of motor performance (i.e., gait) or use of compensatory strategies while not sacrificing a safe gait in response to increased attentional loads during dual task performance (Kim et al., 2017a).

**TABLE 2:** Contents of concurrent dual task used during intervention.

**Type of concurrent rhythm task**	**Level of concurrent task**		
	**1**	**2**	**3**
Playing instruments with different rhythm patterns using both hands	Playing regular beat patterns or playing to the rhythmic cueing at the adjusted tempi (e.g., faster or slower)	Playing combination of different rhythms	Playing different rhythm patterns while shifting from one to another as requested
Rhythmic chanting or singing	Doing simple chanting or singing familiar songs with the use of rhythmic cueing	Doing simple chanting or singing familiar songs in alternation with the investigator	Chanting or singing songs with the rhythmic cueing while memorizing or changing a part of the songs

Furthermore, a previous review concluded that training older populations to shift their attention between tasks may be more effective in increasing dual task intervention outcomes (Agmon et al., 2014). As such, in this study, as the level of the task increased, concurrent rhythm tasks were adjusted to involve a greater number of task-shifting components. Although the number of walking and bimanual tapping tasks was maintained across sessions, the rate of performing these tasks and the points at which tasks were adjusted to the next level depended on each participant.

### Procedures

Each participant in the intervention group received 30-min individual sessions twice a week for a total of 16 sessions over 8 weeks. The rhythm-motor dual task intervention was conducted in a quiet place within their apartment or the community center. The duration of each stage was determined by each participant’s dual task performance. At pretest and post-test, cognitive and balance/mobility measures were administered. Rhythm-motor tasks were also implemented.

### Measures

In this study, cognition, balance/mobility, rhythm-motor dual tasks were measured to examine changes after participation in the intervention. For cognitive measures, Trail Making Test (TMT), and Wisconsin Card Sorting Test (WCST) were measured. The TMT consists of two subtests: TMT-A and TMT-B (Reitan, 1956). During TMT-A, each participant connected numbers sequentially from 1 to 15 and during TMT-B, he/she connected numbers and words for the days of the week alternatively. These tests assess information processing speed, working memory, and cognitive flexibility. While both tests require keeping track of information in a sequence, the TMT-B also involves inhibitory attentional control and set-shifting by requiring the participants to shift their attentions back and forth between two sets of tasks (Arbuthnott and Frank, 2000).

During the WCST, participants are required to sort cards (i.e., based on color, form, or number of figures) in response to feedback from the tester about whether each response is correct (Heaton, 1981). This test assesses the ability to develop problem-solving strategies by measuring the number of correct responses and errors. In addition, the initial sorting principle is later changed to activate each participant’s inhibitory control of attentions, set-shifting, and cognitive flexibility as measured by the test’s index of preservative errors.

For balance/mobility measures, the Timed Up-and-Go (TUG) test was administered. The TUG assesses balance and functional mobility by measuring the time to complete the task of standing up from an arm-chair, walking 3 m away, turning back, and sitting down. For older adults, more than 13.5 s to complete the test indicates high risk of falling (Shumway-Cook et al., 1997).

The Activities-Specific Balance Confidence (ABC) scale consists of 16 items and is self-administered (Powell and Myers, 1995). Participants were asked to rate how confidently they were able to perform presented tasks (e.g., walking around the house, walking up or down stairs, walking outside on icy sidewalks) while maintaining balance and not falling from 0% = “no confidence” to 100% = “completely confident.”

For gait measures, participants were instructed to walk a 6-m hallway within the institution where they were recruited from. Walking tasks were conducted in both the single and dual task conditions. The single task (Walk_single_) was to walk at preferred speed. For dual task measures, two types of rhythm-motor tasks and two traditional cognitive-motor tasks were implemented. Rhythm-motor tasks included matching the rhythmic cueing while walking (Walk_RC_) and striking instruments using both hands while walking (Walk_IP_). Cognitive-motor tasks included the tasks to count forward from a two-digit number by 3 s while walking (Walk_Count.f__3_) and to count backward from a two-digit number by 3 s while walking (Walk_Count.__b__3_).

During such walking tasks, steps and time were measured by an independent assessor within the pre-designated walking distance with initial acceleration and terminal deceleration distances excluded. Then, walking speed and stride length were calculated by dividing the walking distance (meter) by the time (seconds) to walk the distance and by dividing the number of steps by 2 and dividing the distance (meter) by the counted number. For each measure (i.e., walking speed and stride length), the calculated values from two trials of walking were averaged.

In addition, the ratio of stride length to walking speed was measured by dividing the step length (i.e., the stride length divided by 2) by the walking speed. The measure indicates whether the pattern of movement and degree of postural control is associated with safe walking. For example, decreased stride length leads to a lower value in this measure, which is indicative of increased cautiousness while walking and less risk for falling.

### Data Analysis

For all measures, descriptive data (i.e., mean and standard deviation) were collected. For each of outcome domains (cognitive, balance, and gait), correlation analysis was conducted among included measures. For some measures that were found to be correlated strongly with each other, a mixed model of repeated measures multivariate analysis of variance were conducted in order to control for Type I errors that could occur from multiple comparisons. For other measures that were considered independent from each other, a mixed model of repeated measures ANOVA was implemented to compare the measured data at pretest and post-test between the groups by including a within-subject factor of time and a between-subject factor of group.

## Results

For the baseline measured at pretest for both groups, an independent *t*-test was conducted. The results showed no significant differences between groups and accordingly, indicated that both groups exhibited similar level of cognitive functioning, balance/mobility, and gait parameters. The changes in each measure at pretest to post-test are summarized in Table 3. Changes in gait measures, including walking speed and stride length in each single and dual task condition, and the gait ratio are also displayed.

**TABLE 3:** Changes in cognitive, balance, and gait measures in each group.

	**Intervention (*n* = 10)**		**Control (*n* = 10)**	
**Parameter**	**Pretest *M* ± *SD***	**Post-test *M* ± *SD***	**Pretest *M* ± *SD***	**Post-test *M* ± *SD***
**Cognitive measure**				
TMT-A, time (seconds)	24.22 ± 7.25	24.90 ± 12.48	33.97 ± 15.12	25.27 ± 6.77
TMT-B, time (seconds)	63.74 ± 60.62	36.02 ± 15.32	48.41 ± 19.14	103.48 ± 108.18
WSCT correct responses, *n*	35.20 ± 13.72	41.30 ± 14.13	26.00 ± 10.37	24.80 ± 12.60
WSCT errors, *n*	28.80 ± 13.72	22.70 ± 14.13	38.00 ± 10.37	39.20 ± 12.60
WSCT perseverative errors, *n*	18.60 ± 13.86	16.10 ± 11.47	26.50 ± 12.23	23.30 ± 12.64
**Balance measure**				
TUG, time (seconds)	10.92 ± 2.41	9.94 ± 2.41	10.14 ± 2.42	9.66 ± 2.27
K-ABC, score	78.95 ± 14.46	80.00 ± 13.12	76.91 ± 18.19	78.37 ± 14.34
**Gait measure**				
**Walking speed, m/s**				
Walk_single_	1.03 ± 0.31	0.99 ± 0.32	1.06 ± 0.25	1.06 ± 0.20
Walk_RC_	1.07 ± 0.34	0.97 ± 0.37	0.93 ± 0.18	0.98 ± 0.22
Walk_IP_	1.02 ± 0.35	0.93 ± 0.32	0.96 ± 0.21	0.99 ± 0.18
Walk_Count.f3_	0.92 ± 0.36	0.82 ± 0.32	0.89 ± 0.20	0.89 ± 0.22
Walk_Count.__b__3_	0.86 ± 0.36	0.76 ± 0.33	0.82 ± 0.26	0.82 ± 0.23
**Stride length, m**				
Walk_single_	1.10 ± 0.26	1.11 ± 0.26	1.05 ± 0.16	1.07 ± 0.14
Walk_RC_	1.11 ± 0.25	1.06 ± 0.24	0.97 ± 0.11	1.01 ± 0.12
Walk_IP_	1.07 ± 0.29	1.06 ± 0.28	0.99 ± 0.13	1.03 ± 0.11
Walk_Count.f3_	1.11 ± 0.27	1.06 ± 0.28	0.99 ± 0.11	1.02 ± 0.13
Walk_Count.__b__3_	1.05 ± 0.27	1.03 ± 0.26	0.97 ± 0.14	1.01 ± 0.12
**Gait ratio**				
Walk_single_	0.53 ± 0.14	0.57 ± 0.08	0.51 ± 0.05	0.51 ± 0.04
Walk_RC_	0.54 ± 0.12	0.58 ± 0.12	0.53 ± 0.06	0.54 ± 0.10
Walk_IP_	0.54 ± 0.10	0.59 ± 0.09	0.53 ± 0.06	0.53 ± 0.06
Walk_Count.f3_	0.68 ± 0.28	0.69 ± 0.21	0.57 ± 0.09	0.59 ± 0.11
Walk_Count.__b__3_	0.63 ± 0.17	0.74 ± 0.26	0.67 ± 0.31	0.65 ± 0.16

*Data presented as *M* ± *SD*. MMSE, Mini-Mental State Examination; GDS, Geriatric Depression Scale; TMT, Trail Making Test; WCST, Wisconsin Card Sorting Test; TUG, Timed Up and Go test; K-ABC, Korean version of Activities-Specific Balance Confidence scale; RC, rhythmic cueing; IP, instrument playing; Count.f3, Counting forward by 3 s; Count.b3, Counting backward by 3 s.*

When correlation analyses were conducted, strong correlation was found among subtest measures of WCST at a significant level. Accordingly, a mixed-model of repeated measures MANOVA was conducted for such measures. For each of other measures, a mixed model of repeated measures ANOVA was conducted to compare the two groups in terms of changes in each measure across time. The results are displayed in Table 4. There were significant time effects in TUG and gait ratio measured in the condition of walking with instrument playing, indicating that changes in such measures were significant across the time. For TUG, both groups showed decreases in the time to complete the test at post-test. For the gait ratio in the walking with instrument playing, looking at the changes in terms of the mean, the measure tended to increase at post-test. Also, significant group effects were found for the WCST. While the intervention group gave more correct responses following the intervention, the control group gave more incorrect responses. A significant interaction effect for time and group was observed for the TMT-B. While the intervention group exhibited took less time to complete the test at post-test, the control group took more to complete the test. In addition, in terms of gait measures, a significant interaction effect between time and group was found for the gait ratio during the walking with instrument playing condition. While the intervention group showed increased gait ratio, the control group did not show remarkable change in the measure.

**TABLE 4:** The results of a mixed model repeated measures ANOVA.

**Variable**	**Repeated measures results**		
	**Time effect (*t*, *p*)**	**Group effect (*t*, *p*)**	**Time ^∗^ Group (*t*, *p*)**
**Cognitive**			
TMT-A	1.559, 0.228	1.856, 0.190	2.130, 0.162
TMT-B	0.512, 0.484	1.564, 0.227	4.695, 0.044^∗^
WCST^a^	0.452, 0.644	3.854, 0.042	1.673, 0.217
**Balance**			
TUG	7.308, 0.015^∗^	0.271, 0.609	0.825, 0.376
K-ABC	0.119, 0.734	0.104, 0.751	0.003, 0.955
**Gait ratio**			
Walk_single_	2.096, 0.165	1.407, 0.251	1.174, 0.293
Walk_RC_	1.575, 0.225	0.653, 0.430	0.656, 0.429
Walk_IP_	5.615, 0.029^∗^	1.302, 0.067	5.800, 0.027^∗^
Walk_Count.f3_	0.720, 0.407	1.588, 0.224	0.017, 0.896
Walk_Count.__b__3_	1.201, 0.288	0.066, 0.799	3.041, 0.098

*^∗^*p* < 0.05. ^*a*^A mixed model of multivariate analysis of variance.*

## Discussion

**FIGURE 1:**
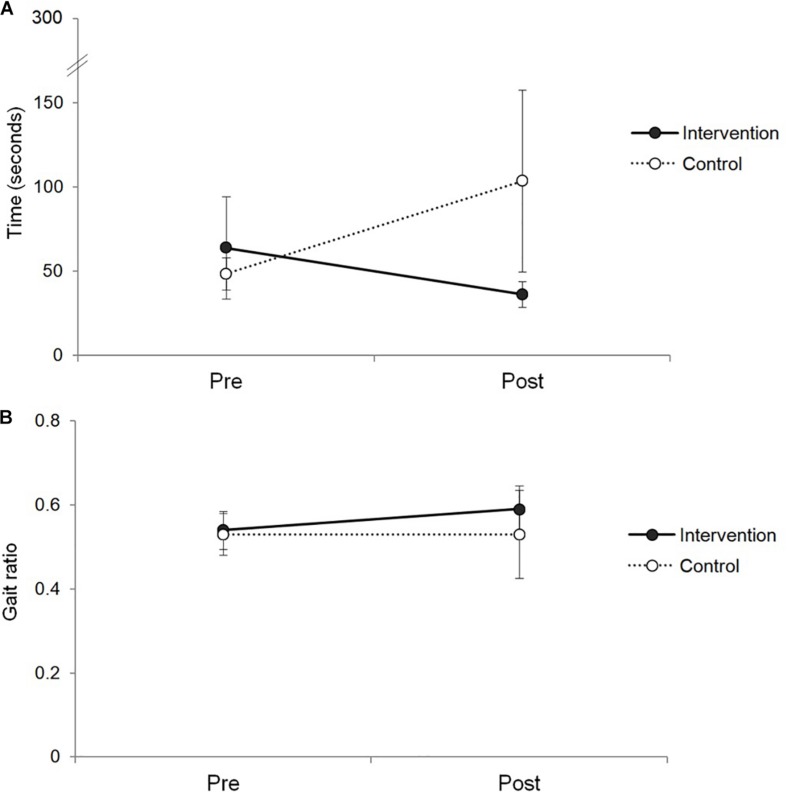
Changes in TMT-B and gait ratio measures in the intervention and control groups. Panel **A** shows the changes in time to complete the TMT-B test, and panel **B** shows the changes in gait ratio measures.

This study aimed to examine changes in cognitive and gait-related measures in healthy older adults after participating in the rhythm-motor dual task intervention. Changes in measured parameters were compared to those of the control group who did not receive the intervention. Among the cognitive measures, a group difference was found for the TMT-B (see Figure 1). While the intervention group spent less time completing the test at post-test, which indicates improved performance on the test, the control group spent more time completing the test. Meanwhile, for TMT-A, there was no significant difference between the groups. While both of the TMT subtests measures attentional control, the TMT-B involves increased cognitive demand and requires cognitive inhibition and set-shifting (Arbuthnott and Frank, 2000). Executive functioning as measured by the TMT-B plays an important role in gait control and that it could be a predictive factor for falls (Ijmker and Lamoth, 2012). Therefore, a significant difference in this measure between groups supports that the rhythm-motor dual task intervention effectively mediated executive control of attention, which would also be a critical factor for gait control.

For the other cognitive measure of executive function (i.e., WCST), the intervention group showed an increase in their number of correct responses at post-test compared to the control group. Age-related decline on this test was found to be alleviated by utilization of external cues (e.g., verbal feedback), resulting in adaptation to requested shifts in cognitive processing (Ashendorf and McCaffrey, 2008). The tendency toward improvement on this task observed in the intervention group supports this intervention’s potential for improving cognitive flexibility and attentional control, particularly cognitive set-shifting.

In terms of the balance/mobility measures, both groups showed decreases in their time to complete the TUG test. For perceived confidence in activity-specific balance, both groups showed increases in their scores at post-test. Although the intervention group showed improvement, so did the control group, and no information about the participations’ level of physical activity outside of the study was available that could help parse the results. It could be suggested that the rhythm-motor dual task intervention was constructed to target fall prevention by primarily addressing cognitive control rather than balance or other physical attributes. Additional studies are needed that consider the influence of current activities that might affect balance and mobility in older adults.

Furthermore, in the study, there was a significant interaction effect between group and time in gait ratio during walking with instrument playing condition. While the intervention group showed increases in this measure, the control group exhibited the opposite (see Figure 1). For each gait parameter, the intervention group showed decreases in both of walking speed and stride length when asked to walk while playing an instrument, which means slower walking with shorter stride length. Meanwhile, the control group increased their walking speed and stride length.

Gait speed has been identified as a strong predictor of falling, and maintaining a constant walking speed in a challenging environment (e.g., increased cognitive demand while walking) is important for a safe gait (Hak et al., 2013). In this study, the intervention group showed decreased walking speed, even in the single walking condition, which interferes with gait stability (Beauchet et al., 2008), It is noteworthy that decreased walking speed of the intervention group at post-test was still in the range of normal speed, given that slower walking speed was identified less than 0.7 m/s in previous studies (Cesari et al., 2005; Verghese et al., 2007). A faster gait (greater than 1 m/s on average) rather indicates decrease in gait control (van Iersel et al., 2006) and accordingly, the changes in walking speed in the intervention group may be attributed partially to enhanced gait control. Also, such decreased walking speed was offset by shorter stride length, which is indicative of cautious walking. Given that slower gait can be offset by a relative decrease in step length (Espy et al., 2010) and combination of decreased step length and increased step frequency can be the way to deal with maintaining gait stability (Hak et al., 2013), the findings of this study suggest that the participants used a compensatory strategy for a safe gait when increased cognitive demands interfered with their gait control.

Changes in gait ratio during the instrument playing condition supports that the participants used their limited cognitive capacity more efficiently during ongoing cognitive-motor dual task conditions. In terms of involved music perception and production, a certain level of cognitive activation and immediate motor activation occurs simultaneously with music stimuli being processed at both the cortical and subcortical levels (Särkämö et al., 2016). Based on previous evidence, it can be predicted that auditory-motor interaction drives cognitive activation resulting in efficient movement planning and execution during instrument playing (Baumann et al., 2005; Zatorre et al., 2007). During instrument playing as a dual task, produced sound from instrument playing and externally provided regular rhythm patterns can activate auditory-motor interaction assisting executive control for walking.

Considering high level of education of the participants in this study, it is meaningful to pay attention to the changes in the executive control measures of the intervention group. Rhythm-motor dual task with adjustment of the tempi and changes in musical behaviors (e.g., singing or chanting) seems to require enhanced cognitive control during intervention. One of the major changes in bimanual task performance in aging is synchronization to external cues at adjusted tempi (Kim et al., 2017a, b), and integration of different tempo and type of movement in the intervention seems to involve and enhance cognitive processing for older adults. As a form of bimanual movement, instrument playing can be effectively applied as challenging but adjustable cognitive contents due to its feedback-feedforward mechanism of musical instrument playing.

Although potential of this rhythm-motor dual task intervention was identified in this study as an exploratory attempt to integrate music-based strategies for cognitive and gait control of older population, the results should be generalized with caution. A small sample size and multiple comparisons were not yet sufficient to corroborate that outcomes of this intervention can be replicated in other populations and settings. Further studies are needed to validate the effects of the intervention, while including increased sample sizes and variables that were found to be more directly influenced by the intervention.

## Conclusion

This study supports the potential of the rhythm-motor dual task intervention for fall prevention of older adults. Comparison of changes in older adults participating in the intervention with those who did not indicates that the intervention can effectively mediate the ability to increase available cognitive resources and utilize their own adaptive strategy for cognitive and gait control. The use of rhythmic cueing and musical tasks was also identified as an effective agent for intervening in motor and cognitive control. Additional studies are needed to confirm the effects of the intervention with a larger sample size. Future research also needs to examine how such improvement can be maintained.

## Data Availability Statement

The datasets generated for this study are available on request to the corresponding author.

## Ethics Statement

All procedures were reviewed and approved by the Institutional Review Board at Ewha Womans University (IRB No. 136-5). The patients/participants provided their written informed consent to participate in this study.

## Author Contributions

SK conceptualized the research idea, received the research fund, analyzed the data, discussed the results, and wrote the manuscript. GY collected and analyzed the data, discussed the results, and wrote the manuscript.

## Conflict of Interest

The authors declare that the research was conducted in the absence of any commercial or financial relationships that could be construed as a potential conflict of interest.
